# Dietary inflammatory index mediation lifestyle patterns and depression among women with osteopenia or osteoporosis

**DOI:** 10.3389/fnut.2025.1578954

**Published:** 2025-07-01

**Authors:** Baoping Wang, Yuxin Fan, Shaofang Tang, Weihong Guo, Yin Li, Chenlin Dai

**Affiliations:** ^1^Department of Endocrinology and Metabolism, Tianjin Medical University General Hospital, Tianjin, China; ^2^Medical School of Tianjin University, Tianjin University, Tianjin, China

**Keywords:** dietary inflammation index, depression, lifestyle patterns, osteopenia, osteoporosis, NHANES

## Abstract

**Objective:**

Lifestyle factors play a critical role in osteoporosis management and are closely linked to the development and progression of comorbid depression. This study examines lifestyle patterns and their association with depression in individuals with osteopenia or osteoporosis, while assessing the mediating role of the Dietary Inflammatory Index (DII).

**Methods:**

Data from the National Health and Nutrition Examination Survey (NHANES) 2009–2020 were analyzed using latent class analysis (LCA) to classify 3,384 adults based on their lifestyle behaviors. A generalized linear model (GLM) evaluated the effects of lifestyle patterns on depression, and mediation analysis tested associations between these patterns, DII, and Patient Health Questionnaire-9 (PHQ-9) scores.

**Results:**

LCA identified three lifestyle groups: healthy but inactive (34.16%), unhealthy (8.78%), and sedentary (57.06%). The unhealthy (OR = 2.848, 95% CI = 1.550–5.234, *p* = 0.001) and sedentary (OR = 1.600, 95% CI = 1.127–2.271, *p* = 0.009) groups were associated with higher depression risk in women. DII partially mediated the relationships between unhealthy lifestyle and PHQ-9 (effect coefficient = 0.095, 95% CI: 0.056–0.135) and between sedentary lifestyle and PHQ-9 (effect coefficient = 0.059, 95% CI: 0.017–0.115).

**Conclusion:**

These findings suggest that lifestyle patterns significantly influence depression in women with osteopenia or osteoporosis, with DII serving as a partial mediator.

## 1 Introduction

Osteoporosis is a chronic systemic skeletal disorder characterized by reduced bone mass and deterioration of bone microarchitecture (1), with its progressive nature posing significant public health challenges (2). Globally, approximately one-third of women and one-fifth of men aged 50 and above develop osteoporosis, exhibiting an estimated prevalence of 18.3% (3, 4). In the United States alone, 14.1 million adults aged 50 and above are currently diagnosed with osteoporosis, with prevalence rates continuing to increase (5, 6). As populations age, the growing number of osteoporosis cases warrants greater focus on its comorbidity with chronic diseases, which substantially compromises patients’ quality of life (7). Bone undergoes dynamic remodeling throughout life, and recent discoveries of its endocrine functions have established the bone–brain axis as a conceptual framework for studying neural function and behavior (8). Bone-derived signaling molecules contribute to both neuropsychiatric disorders, such as depression and neurodegenerative conditions (9). The ICD-11 classifies depression as a syndrome involving distinct clinical symptoms and behavioral manifestations that impair functioning and cause distress (10). In osteoporosis patients, depression correlates with poorer clinical outcomes, including restricted physical activity and unhealthy lifestyle patterns (4, 11).

The relationship between osteoporosis and depression involves complex bidirectional mechanisms, though lifestyle factors play a central moderating role in their development and progression (8, 12, 13). Reduced mobility from osteoporosis often impairs patients’ ability to perform daily activities, while simultaneously isolating them from social support systems, creating conditions that foster maladaptive behaviors (14). These behavioral patterns—including physical inactivity, sleep dysregulation, and substance abuse—collectively contribute to neurobiological changes that elevate depression risk (15–19). Chronic sleep disturbances, for example, alter hormonal homeostasis and amplify inflammatory cascades known to underlie depressive pathophysiology (15). Nicotine exposure from smoking modulates nAChR-mediated neurotransmission, directly affecting neural circuits involved in stress adaptation and mood regulation (16). Alcohol-induced metabolic dysregulation further compounds emotional instability, establishing a self-perpetuating cycle of depressive symptomatology (17). The activity restriction inherent to osteoporosis frequently precipitates a sedentary lifestyle that independently predicts depression onset (18), whereas exercise demonstrates antidepressant effects through neurotrophic and anti-inflammatory mechanisms (19). Existing research has extensively focused on discrete behavioral factors rather than examining the heterogeneous lifestyle profiles that may differentially influence depression risk in osteoporotic populations. Characterizing these multidimensional behavioral patterns could support more precise preventive interventions and treatment approaches.

In osteoporosis management, dietary habits substantially influence bone mineral density and complication risks (20). Patients with osteoporosis often follow pro-inflammatory diets, which may contribute to depression pathogenesis (21, 22). The Dietary Inflammatory Index (DII) quantifies diet-induced inflammation, enabling assessment of its health effects (23). Pro-inflammatory diets—high in saturated fats, trans fats, and refined sugars—correlate with elevated DII scores and depression risk (24), whereas anti-inflammatory diets abundant in omega-3 fatty acids, whole grains, and produce yield lower DII scores and reduced depression incidence (25). These dietary differences regulate bone mass and simultaneously provide nutrients that support mood and cognition. Healthy lifestyles combining physical activity, adequate sleep, and limited alcohol and tobacco use further promote anti-inflammatory dietary patterns. This reciprocal relationship between lifestyle and diet may improve overall health outcomes (26, 27). The DII potentially mediates the association between osteoporosis-related lifestyle factors and depression, indicating that dietary inflammation modulates their link to depressive symptoms.

This study examined the association between lifestyle factors, dietary inflammation, and depression risk in adults with osteopenia or osteoporosis. We assessed lifestyle characteristics in this population, evaluated relationships between lifestyle factors and depression, and determined whether the DII mediates these associations. The DII may differentially influence depression risk depending on distinct lifestyle patterns among individuals with osteopenia or osteoporosis. These findings could guide targeted interventions combining lifestyle adjustments and dietary approaches to reduce the depression burden in this patient group.

## 2 Methods

### 2.1 Study population

The National Health and Nutrition Examination Survey (NHANES) is a cross-sectional, population-based study designed to assess the health status, nutritional habits, and lifestyle of non-institutionalized U. S. civilians. We analyzed NHANES data from three survey cycles (2009–2010, 2013–2014, and 2017–2020). The National Center for Health Statistics (NCHS) provides full documentation of the study design and data collection procedures (28, 29). A total of 14,360 participants aged 20 years or older were identified, and 3,384 participants with complete data entered the final analysis. To account for potential bias introduced by missing values, we retained only complete-case observations in the analysis. The detailed screening process is shown in Supplementary Figure S1.

### 2.2 Assessment of osteopenia and osteoporosis

Bone mineral density (BMD) was measured by dual-energy X-ray absorptiometry (DXA) using QDR 4500A fan-beam densitometers (Hologic, Inc., Bedford, MA, USA). Standard left hip scans provided total BMD values for the femur, femoral neck, and trochanters. The World Health Organization (WHO) defines osteopenia and osteoporosis in adults aged ≥50 years as BMD values that are 1–2.5 standard deviations (SD) or >2.5 SD below the young adult mean (20–29 years), respectively (30). These diagnostic thresholds apply equally to both sexes and collectively indicate low bone density (31). Patients with concurrent comorbidities were excluded from the analysis.

### 2.3 Assessment of lifestyle behavior

Lifestyle behavior data were obtained via in-person questionnaires and 24-h dietary recalls. Five dichotomized lifestyle variables were analyzed: cigarette smoking, alcohol consumption, sleep duration, moderate-to-vigorous physical activity (MVPA), and sedentary behavior. Participants who reported smoking ≥100 cigarettes in their lifetime were classified as unhealthy smokers (coded 1) (32). Unhealthy alcohol consumption was defined as >2 drinks/day for men or >1 drink/day for women, consistent with Dietary Guidelines for Americans (coded 1) (33). Sleep durations <6 h or >8 h, regardless of weekday/weekend patterns, were considered unhealthy (coded 1) (34). According to WHO guidelines, participants who failed to meet the weekly thresholds of 150 min of moderate-intensity activity, 75 min of vigorous-intensity activity, or equivalent combinations were classified as physically inactive (coded 1) (35). Sedentary behavior, defined as >7.5 sitting h/day based on previous studies, was similarly categorized as unhealthy (coded 1) (35, 36).

### 2.4 Assessment of dietary inflammatory index (DII)

The NHANES collected dietary intake data through two 24-h recall interviews, during which participants reported all foods and beverages consumed on the preceding day. Energy and nutrient intakes were derived from these self-reported quantities.

A modified version of the Shivappa et al. (23, 37) methodology was used to calculate the Dietary Inflammatory Index (DII), incorporating 27 dietary components such as macronutrients, vitamins (A, B6, B12, C, D, E), minerals (Fe, Mg, Zn, Se), fatty acids (MUFA, PUFA, n-3, n-6), and bioactive compounds (caffeine, *β*-carotene). Although fewer than 30 nutrients were considered, the DII remained a valid measure (23).

For DII computation, individual nutrient intakes were standardized into *z*-scores by centering them on global mean values and scaling by standard deviations. These *z*-scores were converted to percentiles, symmetrically adjusted to a − 1 to +1 range, and then transformed by doubling, subtracting from 1, and multiplying by nutrient-specific inflammatory weights. The final DII score represented the sum of these weighted values (23). Given the study population’s age (>50 years) and consistent dietary habits, the DII provided a reliable inflammatory assessment.

### 2.5 Assessment of depressive symptoms

The Patient Health Questionnaire-9 (PHQ-9) is a nine-item instrument validated for assessing depressive symptoms based on the Diagnostic and Statistical Manual of Mental Disorders-IV (DSM-IV) criteria. The NHANES used the PHQ-9 to measure depressive symptoms among participants during the 2 weeks preceding the survey (38).

Participants rated the frequency of nine DSM-IV-defined depressive symptoms over this period using a 4-point scale: 0 (“not at all”), 1 (“several days”), 2 (“more than half the days”), or 3 (“nearly every day”). Total scores ranged from 0 to 27, with a threshold of ≥10 suggesting clinically significant depressive symptoms (39).

### 2.6 Covariates

The analysis adjusted for several potential confounders: age, sex, ethnicity (non-Hispanic White, non-Hispanic Black, and others), education level (less than high school, high school or equivalent, and college or above), self-reported household income, and body mass index (BMI). Household income was measured using the poverty–income ratio (PIR) and categorized into high (PIR > 3.5), middle (PIR 1.3–3.5), and low (PIR ≤ 1.3) categories (40). BMI was grouped into normal or low weight (<25.0 kg/m^2^), overweight (25.0–29.9 kg/m^2^), and obese (≥30.0 kg/m^2^) (39).

### 2.7 Data analysis

All data were integrated according to the NHANES protocols, incorporating masked variance and applying the recommended weighting methodology. Latent class analysis (LCA) identified underlying subgroups based on five lifestyle behaviors, with model selection guided by the Akaike information criterion (AIC), Bayesian information criterion (BIC), sample-size adjusted BIC (aBIC), Bootstrap likelihood ratio test (BLRT), and adjusted Lo–Mendell–Rubin test (aLMR). To assess associations between lifestyle patterns and depression in participants with osteopenia or osteoporosis, we employed a generalized linear model (GLM). Mediation analysis further examined relationships among lifestyle patterns, dietary inflammatory index (DII), and PHQ-9 scores in this population. LCA was performed using MPlus 8.3, while mediation analyses utilized IBM SPSS 26.0 with PROCESS Macro Model 4. The remaining statistical procedures were conducted in R 4.2.2, with statistical significance set at a *p*-value of < 0.05 (two-tailed).

## 3 Results

### 3.1 Demographics

Table 1 presents the baseline characteristics of participants with osteopenia and osteoporosis from the NHANES 2009–2010, 2013–2014, and 2017–2020 cycles. The study population comprised 3,384 individuals with a mean (SE) age of 66.14 (9.32) years. Participants showed a mean (SE) DII score of 1.30 (1.99), while 302 individuals (8.92%) exhibited depressive symptoms.

**Table 1:** Basic demographic characteristics.

Variables	Number	*N* (%)/SD
Sex		
Male	1,572	46.45
Female	1812	53.55
Age (year)		66.14 ± 9.32
Ethnicity		
Non-Hispanic White	1834	54.20
Non-Hispanic Black	526	15.54
Other	1,024	30.26
Education level		
Less than high school	738	21.81
High school or equivalent	1789	52.87
College graduate or above	857	25.32
PIR		
Low	918	27.13
Middle	1,320	39.01
High	1,146	33.86
BMI		
Normal or low weight	960	28.37
Overweight	1,593	47.07
Obesity	831	24.56
DII		1.30 ± 1.99
Depression		
Yes	302	8.92
No	3,082	91.08

SD, standard deviation; PIR, poverty–income ratio; BMI, body mass index; DII, dietary inflammatory index.

### 3.2 Latent class analysis of lifestyle behavior

Supplementary Table S1 displays the LCA model results, including fit indices for solutions ranging from 1 to 5 classes. The 4-class model showed non-significant LMR and BLRT values, along with one class comprising less than 5% of the sample, while the 3-class solution demonstrated superior interpretability and the lowest AIC, BIC, and aBIC values.

**Figure 1:**
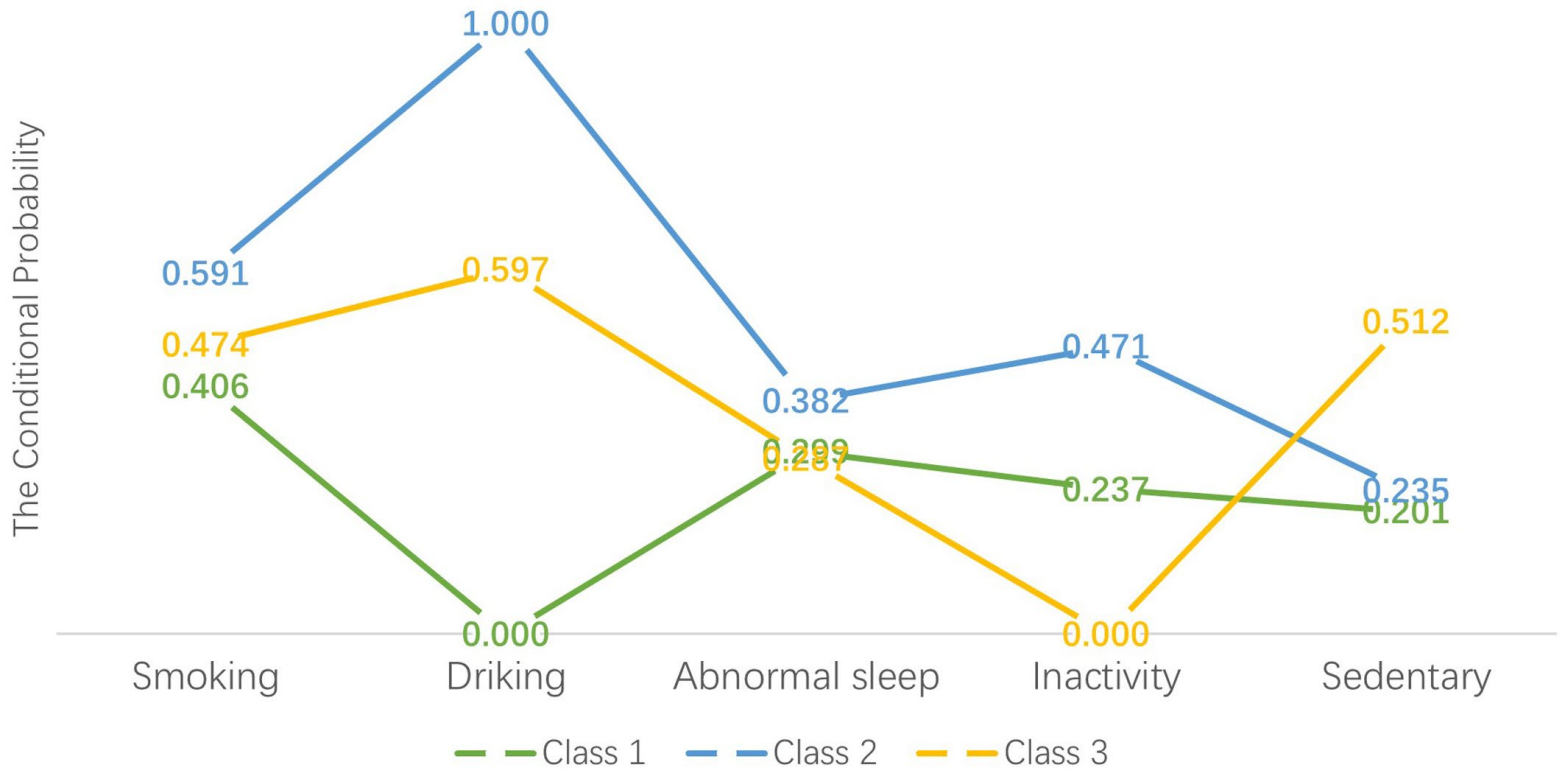
Item-response probabilities of lifestyle behaviors by the three latent class groups. “Healthy but inactivity” group (Class 1) represented 34.16% of the sample (*n* = 1,156). “Unhealthy” group (Class 2) accounted for 8.78% of the full sample (*n* = 297). “Sedentary” group (Class 3) represented 57.06% of the sample (*n* = 1,931).

Figure 1 illustrates the conditional probabilities of lifestyle behaviors across classes. Class 1 exhibited relatively low probabilities for smoking (40.6%), alcohol consumption (0%), and sedentary behavior (20.1%), contrasting with Classes 2 and 3. Class 2 displayed notably high probabilities for smoking (59.1%), alcohol consumption (99.9%), sleep disturbances (38.2%), and physical inactivity (47.1%). Although Class 3 showed the highest sedentary behavior probability (51.2%), its probabilities for sleep disturbances (28.7%) and inactivity (0%) remained lower than other classes. Based on these patterns, we classified Class 1 (*n* = 1,156, 34.16%) as “healthy but inactive,” Class 2 (*n* = 297, 8.78%) as “unhealthy,” and Class 3 (*n* = 1,931, 57.06%) as “sedentary.”

### 3.3 Univariate analysis among lifestyle patterns

Sex, age, ethnicity, and educational level differed significantly across the three lifestyle behavior patterns (sex: *χ*^2^ = 147.700, *p* < 0.001; age: *F* = 26.375, *p* < 0.001; ethnicity: *χ*^2^ = 83.386, *p* < 0.001; education: *χ*^2^ = 30.288, *p* < 0.001), as detailed in Supplementary Table S2.

### 3.4 Effects of lifestyle patterns and DII on depression

After adjusting for age, ethnicity, educational level, PIR, and BMI, women with osteopenia or osteoporosis in the unhealthy (OR = 2.848, 95% CI = 1.550–5.234, *p* = 0.001) and sedentary (OR = 1.600, 95% CI = 1.127–2.271, *p* = 0.009) groups exhibited higher depression risk than those in the healthy but inactive group (Table 2).

**Table 2:** Results of a generalized linear models of lifestyle patterns on depression.

Variables	Unhealthy group Class 2 (Refer to Class 1)		Sedentary group Class 3 (Refer to Class 1)	
	OR (95%CI)	*p* value	OR (95%CI)	*p* value
Depression ^a^	**1.790 (1.149, 2.789)**	**0.010** ^*^	**1.458 (1.095, 1.941)**	**0.010** ^*^
Sex ^b^				
Male	1.165 (0.592, 2.292)	0.659	1.216 (0.728, 2.030)	0.455
Female	**2.848 (1.550, 5.234)**	**0.001** ^*^	**1.600 (1.127, 2.271)**	**0.009** ^*^

CI, confidence interval.

^a^Models adjusted for age, sex, ethnicity, educational level, PIR, and BMI.

^b^Models adjusted for age, ethnicity, educational level, PIR, and BMI.

*Significant correlation, *p* < 0.05.Bold means statistically significant results.

DII scores (*H* = 7.675, *p* = 0.022) differed significantly across the three lifestyle behavior patterns among women (Supplementary Table S3). Higher DII scores were associated with increased depression risk (OR = 1.127, 95% CI = 1.051–1.210, *p* < 0.001) (Supplementary Table S4).

### 3.5 Lifestyle patterns, DII, and PHQ-9 scores

**Figure 2:**
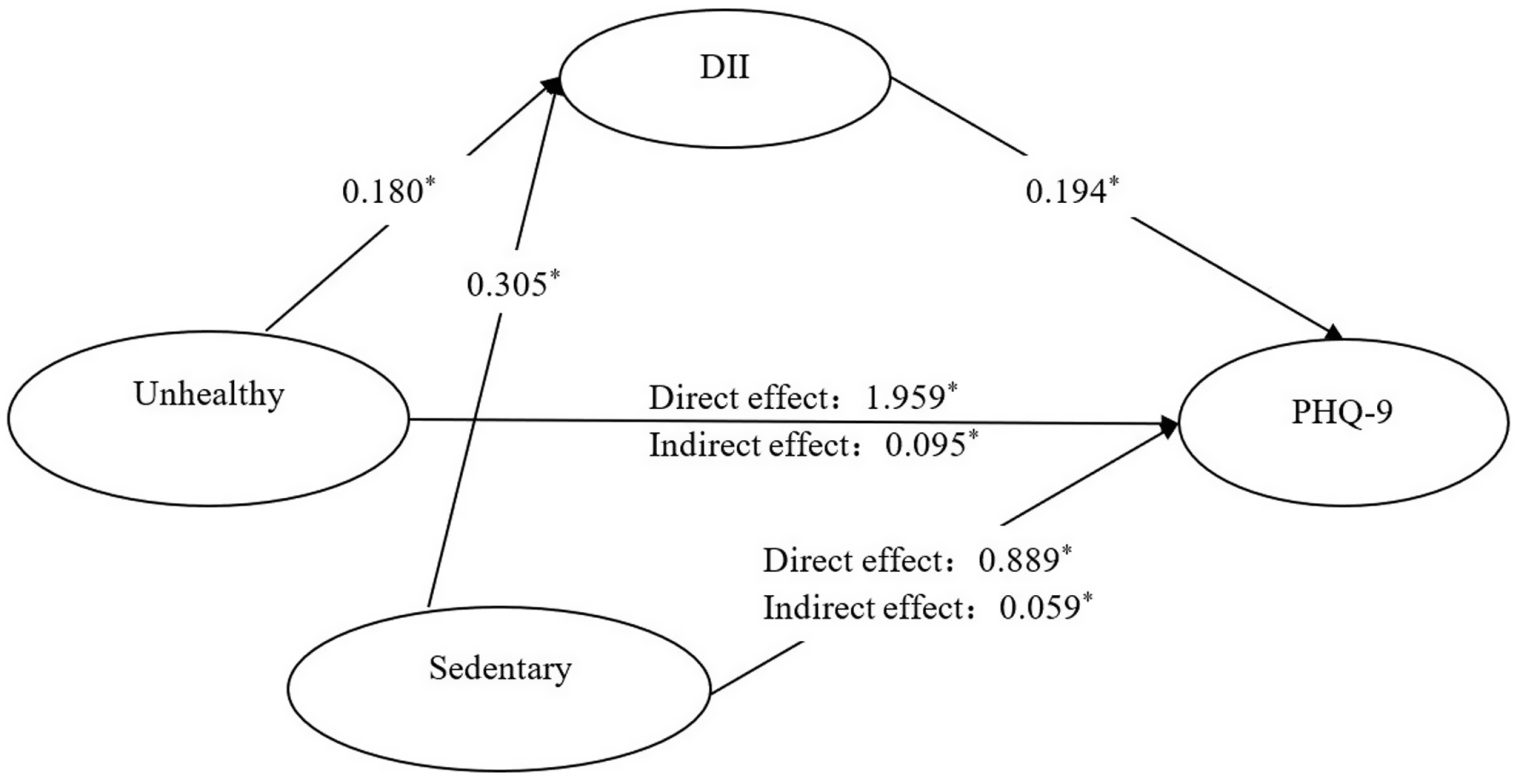
Mediating effect of DII in the relationship between lifestyle patterns and PHQ-9 among adults with osteopenia or osteoporosis. *: *p* < 0.05; coefficients have been standardized; Reference group: Healthy but inactivity group. Path 1: Unhealthy group→DII → PHQ-9. Path 2: Sedentary group→DII → PHQ-9.

DII, lifestyle patterns, and PHQ-9 scores showed significant intercorrelations in women with osteopenia or osteoporosis (Supplementary Table S5). Mediation analysis revealed that DII partially accounted for the associations between both unhealthy lifestyle patterns and PHQ-9 scores (*β* = 0.095, 95% CI: 0.056–0.135) and sedentary behavior and PHQ-9 scores (β = 0.059, 95% CI: 0.017–0.115). These mediation effects corresponded to 4.63 and 6.22% of the total associations between lifestyle factors and depressive symptoms, respectively (Supplementary Table S6; Figure 2).

## 4 Discussion

Our analysis of lifestyle behaviors in adults with osteopenia and osteoporosis revealed three distinct patterns: healthy, unhealthy, and sedentary, each characterized by at least one unhealthy behavior. The distribution of these patterns varied across demographic groups, with osteoporosis and osteopenia patients predominantly clustered in the sedentary group (60.75% of men and 53.86% of women). Men were significantly overrepresented in the unhealthy group compared to women (13.61% versus 4.58%), which was consistent with established gender disparities in smoking and alcohol consumption (41, 42), although alcohol consumption has increased among women in recent years. Participant age differed markedly among the groups, with the lowest mean age in the healthy but inactive group and the highest in the unhealthy group, reflecting the known age-related accumulation of risk behaviors that may accelerate chronic disease development (43). As anticipated, the unhealthy group showed the highest mean DII scores, while the healthy but inactive group displayed the lowest, corroborating evidence linking healthy lifestyles with anti-inflammatory diets (26, 27). These results demonstrate how lifestyle factors interact synergistically to influence health outcomes (44). The high frequency of concurrent unhealthy behaviors in our cohort underscores the need for integrated interventions targeting multiple risk factors simultaneously, which could substantially improve overall health and reduce comorbidity burdens in this population.

Significant disparities emerged in depression risk associated with lifestyle factors, particularly among women with osteopenia or osteoporosis. This finding raises critical questions about potential gender differences in the pathways linking these conditions to comorbid depression. Our study focused exclusively on adults aged 50 and above, particularly women, who typically undergo perimenopause—a period marked by hormonal fluctuations that may simultaneously increase susceptibility to both bone loss and depression (45). The anti-inflammatory properties of estrogen suggest a possible mechanism, as declining levels in postmenopausal women could exacerbate depression through inflammatory pathways (46). Gender-specific neuroendocrine stress responses may further contribute to this phenomenon (47). Depression also manifests differently between genders (48), with women generally experiencing more severe and persistent symptoms than men (49). Notably, lifestyle factors interact with depression risk in a gender-dependent manner: Fan et al. demonstrated stronger associations between tobacco smoke exposure and depressive symptoms in women than men (50), while sleep patterns disproportionately affect women’s mental health (51). These observations position gender as a key modifier of depression risk, particularly regarding lifestyle interventions. Women with osteopenia or osteoporosis may therefore derive greater mental health benefits from lifestyle improvements, whereas additional factors—including independent effects or behavioral interactions—likely influence outcomes in men. Further investigation should clarify the complex interplay between lifestyle factors, mediating mechanisms, and depression development in this patient population.

Our findings reveal the unique role of the DII in linking lifestyle factors to depression, demonstrating the need for targeted interventions. Among women with osteopenia or osteoporosis, the DII partially mediated associations between unhealthy dietary patterns and PHQ-9 scores, as well as between sedentary behavior and depressive symptoms. These observations imply that dietary interventions reducing inflammation could effectively mitigate depression risk in this population. Shifting toward anti-inflammatory diets while minimizing pro-inflammatory food intake may substantially decrease PHQ-9 scores and depression incidence. However, integrating dietary modifications with broader lifestyle interventions targeting multiple risk factors may yield greater benefits for depressive symptom relief. The pronounced mediating effect of the DII underscores the critical role of dietary management—particularly anti-inflammatory eating patterns—in alleviating depression among osteopenic and osteoporotic patients. This finding corroborates existing evidence that anti-inflammatory diets positively influence mental health (26, 27), suggesting that such diets may buffer depression’s effects on inflammatory markers (52). Chronic low-grade inflammation has been implicated in psychiatric and neurodegenerative disorders. Research consistently shows that diets rich in sugar, refined carbohydrates, saturated fats, and processed meats exacerbate inflammation and are associated with cardiovascular disease, diabetes, cancer, and cognitive impairment. Recent studies further connect these pro-inflammatory diets with elevated depression risk in adults (53). Since proper neurotransmitter synthesis requires adequate amino acids, minerals, and vitamins, nutritional deficiencies may simultaneously affect depression susceptibility and HPA axis function (54). However, the incomplete mediation observed suggests that additional pathways beyond inflammation alone may be involved. 25-Hydroxyvitamin D is not only significantly associated with BMD but also potentially with symptoms of depression (55). In addition, the role of vitamin D in neuroendocrine regulation and bone homeostasis should not be ignored (56). Future research needs to explore its regulatory pathways further, particularly in conjunction with vitamin D receptor gene polymorphisms. Other potential pathways of influence include gut flora, which may be involved in the co-morbid mechanisms of depression and bone loss through immune regulation (e.g., Th17/Treg balance), short-chain fatty acid metabolism, and other related pathways. A clinical study found that the gut microbiota of patients with inflammatory depression exhibited a higher level of *Anaplasma* spp. and a lower level of *Clostridium* spp. and an increase in butyrate metabolism of abnormal SCFA-producing bacteria (57). Longitudinal investigations are needed to clarify these complex relationships and assess the effectiveness of tailored dietary and lifestyle interventions for individuals with osteopenia and osteoporosis. Additionally, clinical trials must establish optimal implementation strategies to translate these findings into improved mental and physical health outcomes.

To our knowledge, this study provides the first examination of how lifestyle patterns and dietary inflammation jointly influence depression risk in adults with osteopenia or osteoporosis. The results reveal distinct mediating effects of the Dietary Inflammatory Index (DII) in this relationship, highlighting the need for targeted interventions. The large, nationally representative NHANES sample strengthens the reliability of these findings and their applicability to U. S. adults with compromised bone health. Several limitations warrant consideration. First, the DII calculation incorporated only 27 food parameters due to data constraints, potentially limiting the assessment’s thoroughness regarding dietary inflammation. Second, while validated instruments were used, the dependence on self-reported dietary recalls and PHQ-9 depression screening introduces potential recall bias. Although depressive states were categorized using established criteria, future work should use more nuanced analytical methods. Third, the cross-sectional design prevents causal inference, underscoring the need for longitudinal studies to clarify temporal relationships among these factors. Future research should broaden the scope to include individuals across the bone density spectrum, even without clinical diagnoses, to better inform prevention efforts. Additionally, our findings require validation in non-U. S. populations. Finally, unmeasured medication use may have confounded the observed associations between depression and dietary behaviors.

## 5 Conclusion

Adults with osteopenia or osteoporosis typically demonstrate lifestyle patterns involving at least one unhealthy behavior. These patterns correlate with increased depression risk in women. The Dietary Inflammatory Index mediates this relationship through multiple pathways, highlighting the need for population-specific interventions that account for varying lifestyle profiles.

## Data Availability

The publicly available data sets used in this study can be found at: https://wwwn.cdc.gov/nchs/nhanes/default.aspx.
