# Supplementary material for: Dietary inflammatory index mediation lifestyle patterns and depression among women with osteopenia or osteoporosis
Source: Front Nutr. 2025 Jul 1;12:1578954. doi: 10.3389/fnut.2025.1578954 (PMC12259418; doi:10.3389/fnut.2025.1578954)
Supplement: Supplementary file 1 [file Data_Sheet_1.pdf]

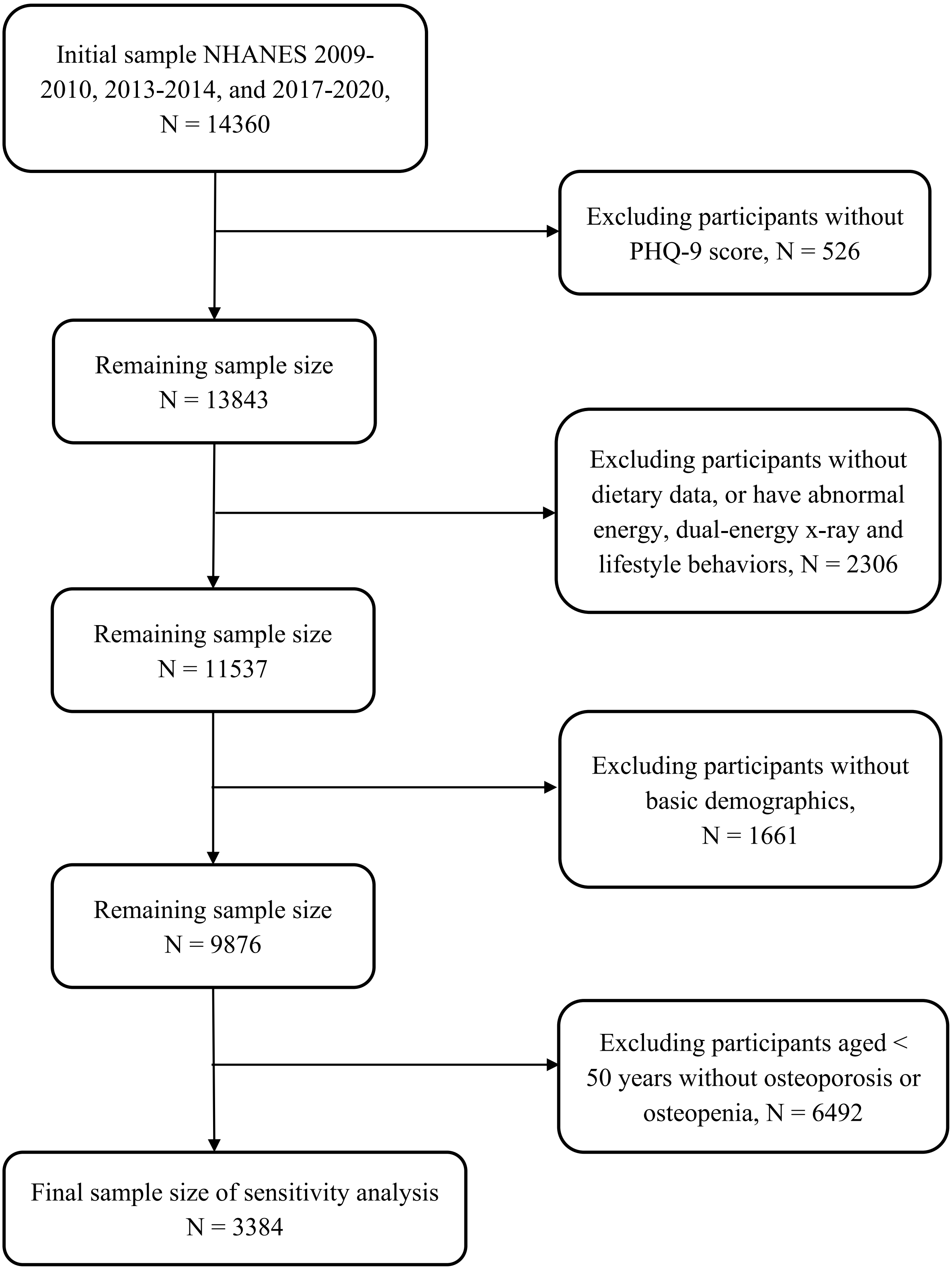

Figure S1 Flow chart for screening of research subjects.

Table S1 Fitting indicators for the latent class analysis.

Number	AIC	BIC	aBIC	entropy	LMR_P	BLRT_P	MINOR%
1	20942.706	20973.340	20957.453	1.000			
2	20869.920	20937.315	20902.363	1.000	<0.001	<0.001	15.40%
3	20822.155	20926.311	20872.294	0.958	<0.001	<0.001	8.78%
4	20829.635	20970.552	20897.470	0.666	0.614	0.999	1.15%
5	20836.641	21014.319	20922.172	0.592	0.318	0.667	2.01%

Note: AIC, Akaike Information Criterion; BIC, Bayesian Information Criterion; aBIC, Adjusted Bayesian Information Criterion; BLRT, Bootstrap Likelihood Ratio Test; aLMR, Adjusted Lo-Mendell-Rubin Likelihood Ratio Test. MINOR% is the Minimum Number of Categories percentage (%).

Table S2 Influence of latent class distribution on lifestyle patterns.

Variables	Class 1	Class 2	Class 3	χ^2/ F/ H	p value
Sex [a]				147.700	<0.001*
Male	403	214	955		
Female	753	83	976		
Age (year) [b]	62.46 ± 8.55	66.26 ± 9.22	66.63 ± 9.37	26.375	<0.001*
Ethnicity [a]				83.386	<0.001*
Non-Hispanic White	560	165	1109		
Non-Hispanic Black	134	54	338		
Other	462	78	484		
Education Level [a]				30.288	<0.001*
Less than high school	233	80	425		
High school or equivalent	594	178	1017		
College graduate or above	329	39	489		
PIR [a]				7.221	0.125
Low	293	92	533		
Middle	473	118	729		
High	390	87	669		
BMI [a]				5.132	0.274
Normal or low weight	329	94	537		
Overweight	559	124	910		
Obesity	268	79	484		
DII [c]	1.14 ± 1.90	1.25 ± 2.05	1.35 ± 1.98	4.290	0.117

Depression [a]				8.951	0.011*
Yes	84	37	181		
No	1072	260	1750		

Note: a: chi-square test; b: One-way ANOVA; c: Kruskal-Wallis H test; PIR, poverty income ratio; BMI, body mass index; DII, dietary inflammatory index.

Table S3 Influence of DII distribution on life-style behaviour patterns.

Variables	Class 1	Class 2	Class 3	*H*	*p value*
DII	1.14 ± 1.90	1.25 ± 2.05	1.35 ± 1.98	4.290	0.117
Sex					
Male	0.84 ± 2.01	0.96 ± 1.84	0.93 ± 2.01	0.374	0.829
Female	1.47 ± 2.04	1.61 ± 1.99	1.77 ± 1.86	7.675	**0.022**[*]

Notes: *Significant correlation, P<0.05; DII, dietary inflammatory index.

Table S4 Results of generalized linear models of DII on depression.

Variables	OR (95%CI)	*p* value
DII	**1.127 (1.051, 1.210)**	**<0.001***

Notes: CI: confidence interval, *Significant correlation, P<0.05; DII, dietary inflammatory index.

Models adjusted for age, sex, ethnicity, education level and PIR.

Table S5 Correlation matrix between variables among adults with osteopenia or osteoporosis.

	1	2	3	4	5	6	7	8
Male								
1. Age	–							
2. Ethnicity	−0.251**	–						
3. Education level	0.011	−0.151**	–					
4. PIR	-0.006	−0.151**	0.469**	–				
5. BMI	0.034	−0.055*	−0.019	0.074**	–			
6. PHQ-9	-0.024	−0.059*	−0.109**	−0.162**	0.02	–		
7. Lifestyle patterns	0.130**	-0.023	−0.072**	−0.064*	-0.002	0.114**	–	
8. DII	0.062*	0.024	−0.214**	−0.188**	0.037	0.071**	0.012	–
Female								
1. Age	–							
2. Ethnicity	−0.228**	–						
3. Education level	−0.080**	−0.161**	–					
4. PIR	−0.058*	−0.149**	0.421**	–				
5. BMI	0.019	0.017	−0.127**	−0.103**	–			
6. PHQ-9	−0.083**	0.014	−0.169**	−0.182**	−0.098**	–		
7. Lifestyle patterns	-0.038	−0.183**	0.02	0.043	0.032	0.122**	–	
8. DII	0.051*	0.001	−0.178**	−0.148**	−0.135**	0.055*	0.065**	–

Notes: *Significant correlation, P<0.05; **, P<0.01; PIR, poverty income ratio; BMI, body mass index; DII, dietary inflammatory index.

Table S6 The direct and indirect effects of lifestyle patterns on PHQ-9 among adults with osteopenia or osteoporosis.

Variables		β	Boot SE	Boot CI	
				Lower Limit	Upper Limit
Male					
Class 2 [a]					
	Direct effect	0.255	0.318	−0.368	0.878
	Indirect effect	-0.012	0.029	-0.074	0.045
Class 3 [a]					
	Direct effect	**0.646***	0.221	0.211	1.080
	Indirect effect	-0.010	0.021	-0.055	0.031
Female					
Class 2 [a]					
	Direct effect	**1.959***	0.516	0.947	2.971
	Indirect effect	**0.095***	0.048	0.056	0.135
Class 3 [a]					
	Direct effect	**0.889***	0.216	0.465	1.313
	Indirect effect	**0.059***	0.025	0.017	0.115

Notes: *: P<0.05; a: Reference group: Class 1; Boot SE, bootstrap standard error; CI, Confidence Interval; Boot CI, bootstrap CI. All models were adjusted for age, ethnicity, education level, PIR and BMI.
